# Non-Canonical Replication Initiation: You’re Fired!

**DOI:** 10.3390/genes8020054

**Published:** 2017-01-27

**Authors:** Bazilė Ravoitytė, Ralf Erik Wellinger

**Affiliations:** 1 Nature Research Centre, Akademijos g. 2, LT-08412 Vilnius, Lithuania; bazilerav@gmail.com; 2 CABIMER-Universidad de Sevilla, Avd Americo Vespucio sn, 41092 Sevilla, Spain

**Keywords:** replication control, RNA:DNA hybrid, transcription-initiated replication

## Abstract

The division of prokaryotic and eukaryotic cells produces two cells that inherit a perfect copy of the genetic material originally derived from the mother cell. The initiation of canonical DNA replication must be coordinated to the cell cycle to ensure the accuracy of genome duplication. Controlled replication initiation depends on a complex interplay of *cis*-acting DNA sequences, the so-called origins of replication (*ori*), with *trans*-acting factors involved in the onset of DNA synthesis. The interplay of *cis*-acting elements and *trans*-acting factors ensures that cells initiate replication at sequence-specific sites only once, and in a timely order, to avoid chromosomal endoreplication. However, chromosome breakage and excessive RNA:DNA hybrid formation can cause break-induced (BIR) or transcription-initiated replication (TIR), respectively. These non-canonical replication events are expected to affect eukaryotic genome function and maintenance, and could be important for genome evolution and disease development. In this review, we describe the difference between canonical and non-canonical DNA replication, and focus on mechanistic differences and common features between BIR and TIR. Finally, we discuss open issues on the factors and molecular mechanisms involved in TIR.

## 1. Origin-Dependent Replication

### 1.1. Chromosomal DNA Replication Initiation in Escherichia coli and Saccharomyces cerevisiae

**Figure 1:**
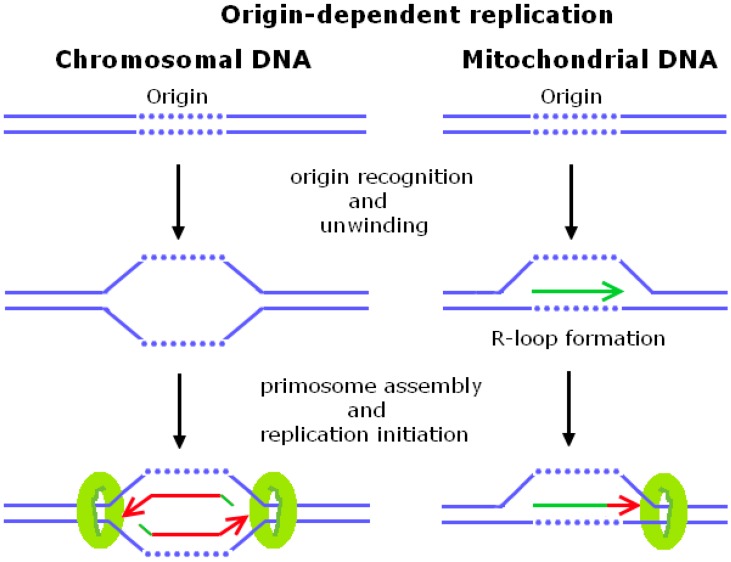
Schematic outline of origin-dependent initiation of chromosomal and mitochondrial DNA replication. *cis*-acting origin DNA sequences (dotted lines), RNA (green), newly synthesized DNA (red), and helicases (green circle) are indicated. Note that chromosomal origin unwinding is driven by protein–DNA interactions, while transcription-dependent R-loop formation is a key step in mitochondrial origin-unwinding. See text for more details.

Replication initiation at a single origin (*ori*) in the bacteria *Escherichia coli* has been the first, and until present, best-described mechanism of a classical replication initiation (see Figure 1; for reviews, see References [1,2,3,4,5]). Within the circular *E. coli* chromosome [6], a single origin called *oriC* provides a platform for protein recognition, local double-stranded DNA (dsDNA) opening, and access of the replication machinery [1]. *OriC* contains multiple repeats of the DnaA-box consensus sequence, and an AT-rich DNA-unwinding element (DUE) adjacent to the DnaA box [7] for the ATP-driven binding of the initiator protein DnaA [1]. *OriC* activation is coupled with bacterial growth rate [8], to efficiently initiate replication at the appropriate time and to avoid replication initiation at particular origins more than once [9,10,11,12,13]. DnaA binds to *oriC* and facilitates binding of the helicase loader-helicase DnaC–DnaB complex to form the pre-priming complex [4,14]. The DnaB helicase then stably interacts with the DnaG primase until RNA primer synthesis is accomplished [15]. Probably, RNA primer synthesis induces conformational changes that release DnaB from DnaG, because primer synthesis is coordinated with or followed by translocation of DnaB to the junction of the replication fork (reviewed in [16]). Subsequently, primer elongation by the DNA polymerase III (DNA Pol III) holoenzyme marks the switch from replication initiation to elongation [17,18]. In contrast to the single origin found in *E. coli*, the budding yeast *Saccharomyces cerevisiae* contains about 400 replication origins. The number of origins per genome is related to the genome size, explaining why eukaryotic genomes require more replication origins for their timely genome duplication [19]. Yeast continues to be one of the most advantageous model systems to study the basis of eukaryotic replication, but in contrast to prokaryotic cells, yeast chromosomes are packaged into nucleosomes. Dependent on their activation timing, replication origins can be separated into early and late replicating origins ([20,21,22], reviewed in [23]). In general, origin-dependent replication initiation requires the following conditions to be fulfilled: recognition of origins, pre-replicative complex (pre-RC) assembly during G1 phase (origin-licensing), and activation of the pre-RC at G1/S-phase (origin-firing; see Figure 1 and Table 1). *S. cerevisiae* origins are defined by a specific consensus sequence, known as autonomously replicating sequence (ARS) [24,25,26]. The AT-rich ARS consensus sequence (ACS) itself is not sufficient for replication initiation [27] but is required for the loading of the pre-RC during G1 phase ([28,29]). The pre-RC is composed of the origin recognition complex proteins Orc1–6 (ORC), Cdc6, Cdt1, and an inactive form of the replicative helicase Mcm2–7 complex ([30,31,32], reviewed in [33]). At G1/S-phase, the Dbf4-dependent kinase (DDK) and S-phase-dependent cyclin-dependent kinases (S-CDKs) phosphorylate Mcm4, Sld2, and Sld3 ([34,35]), prior to the stepwise recruitment of replication factors Cdc45/Sld3/Sld7 and Sld2/Dpb11/Mcm10/GINS/DNA Pol-ε ([36,37,38,39], see [40] for a review). Building up of the active Cdc45/Mcm2–7/GINS (CMG) helicase complex completes the replisome formation [41] and, consequently, DNA synthesis by the DNA Pol-α-primase complex is initiated [42]. Replication initiation is completed by the loading of the proliferating cell nuclear antigen (PCNA) onto the DNA Pol-α synthesized primer to switch to processive DNA synthesis by DNA Pol-ε and Pol-δ (see [43]).

Yeast has developed sophisticated mechanisms to avoid endoreplication events caused by replication re-initiation of already replicated origins. B-type CDKs prevent re-initiation through multiple overlapping mechanisms, including phosphorylation of ORC factors [44], nuclear exclusion of the Mcm2–7 complex and Cdc6 [45,46], transcriptional downregulation, polyubiquitination, and degradation of phosphorylated Cdc6 ([47,48,49]). Under certain conditions, traces of non-phosphorylatable Cdc6 [50] or mutations in components of the pre-replicative complex (origin recognition complex, Cdc6, and MCM proteins are sufficient to re-initiate DNA replication in G2/M cells. In the latter case, a Mec1 and Mre11-Rad50-Xrs2 (MRX) complex-dependent DNA damage signaling pathway is activated to restrain the extent of re-replication and to promote survival when origin-localized replication control pathways are abrogated [51]. Genome-wide analysis suggests that replication re-initiation in G2/M phase primarily occurs at a subset of both active and latent origins, but is independent of chromosomal determinants that specify the use and timing of these origins in S phase [52]. Moreover, the frequency and locations of re-replication events differ from the S to the G2/M phase, illustrating the dynamic nature of DNA replication controls [52]. Additional mechanisms may exist to prevent chromosomal re-replication in metazoans [53]. Interestingly, a recent study identified 42 uncharacterized human genes that are required to prevent either DNA re-replication or unscheduled endoreplication [54].

### 1.2. Mitochondrial DNA Replication Initiation

The variation in mitochondrial DNA (mtDNA) copy number reflects the fact that its replication cycle is not coupled with S phase-restricted, chromosomal DNA replication. Replication of mtDNA is connected with mtDNA transcription through the formation of a RNA:DNA hybrid that has been first detected by electron microscopy as a short three-stranded DNA region [55]. During transcription, the nascent transcript behind an elongating RNA polymerase (RNAP) can invade the double stranded DNA duplex and hybridize with the complementary DNA template strand. The formation of an RNA:DNA hybrid, opposite to an unpaired non-template DNA strand, results in a so-called R-loop structure (for a review see [56]). RNA:DNA hybrids are also the onset of Okazaki fragments, which serve as primers during DNA lagging-strand replication (for a review see [57]; see Figure 1 and Table 1). In the case of mtDNA replication, an R-loop is required for replication priming [58] at the mtDNA heavy-strand replication origin (*OriH*) and light-strand replication origin (*OriL*) [59]. *OriH* and *OriL* consist of a promoter and downstream conserved sequences with a high GC content, and are conserved from *S. cerevisiae* to humans [60]. Budding yeast contains about eight *OriH*-like regions (*ori1–8*; [60]) of which *ori1–3* and *ori5* represent bona fide origins of replication (see [61,62]). The *OriH* region of many organisms includes three conserved sequence blocks called *CSB1*, *CSB2,* and *CSB3* [58], and transition from RNA to DNA synthesis is thought to happen at *CSB2* [63]. Yeast mitochondrial RNA polymerase Rpo41, the helicase Irc3, and the single-stranded DNA (ssDNA)-binding protein Rim1 are the main factors involved in DNA strand separation during mtDNA replication [64,65,66]. After processing by RNase H1, the RNA molecule is used as a primer for DNA synthesis by the *MIP1* encoded mitochondrial DNA polymerase γ (DNA Pol-γ) in budding yeasts [59]. Interestingly, in the absence of RNase H1, primer retention at *OriL* provides an obstacle for DNA Pol-γ [67], leading to mtDNA depletion and embryonic lethality in mice [68].

Apart from DNA Pol-γ, in metazoans the replicative mtDNA helicase Twinkle and the mitochondrial single-stranded DNA-binding protein (mtSSB) play key roles mtDNA replication fork progression (reviewed in [69,70]). The mechanism of mtDNA replication is not fully understood, and various possible mechanisms have been proposed ([71], reviewed in [72]). Currently, there are three main models of mtDNA replication. One is the initial “strand-displacement model”, proposing that leading strand DNA synthesis begins at a specific site and advances approximately two-thirds of the way around the molecule before DNA synthesis is initiated on the lagging strand [73]. A second “strand-coupled model” refers to a strand-asynchronous, unidirectional replication mode [74]. A third “RITOLS model” (RNA incorporation throughout the lagging strand) proposes that replication initiates in the major noncoding region at *OriH*, while *OriL* is a major initiation site of lagging-strand DNA synthesis but the lagging strand is laid down initially as RNA [75]. The idea of transcription-dependent mtDNA replication initiation has been unanimously accepted. However, by taking advantage of mutants devoid of the mitochondrial RNA polymerase Rpo41, Fangman et. al. suggested that replication priming by transcription is not the only mechanism for mtDNA replication initiation in yeast [76,77,78]. Alternatively, the mitochondrial *ori5* has been shown to initiate mtDNA amplification by a rolling circle mechanism [79]. These kinds of replication events are linked to increased mtDNA damage and breaks by oxidative stress, and can be modulated by nuclease and recombinase activities carried out by Din7 and Mhr1, respectively [80].

Collectively, these findings demonstrate that mtDNA replication initiation is capable of adapting to stress situations, and that the stress-dependent, mitochondrial import of nuclear-encoded proteins such as Din7 and Mhr1 could provide another layer of mtDNA replication control. Interestingly, all other proteins involved in replication initiation are nuclear-encoded, and some genes, such as *RNH1*, encode both nuclear and mitochondrial protein isoforms [81]. It will be exciting to see if new players in mtDNA replication initiation may appear in response to different endogenous or exogenous stimuli. To date, little is known about how nuclear and mitochondrial replication checkpoints are interconnected, and how they control mtDNA replication initiation. Interestingly, a recent study showed that the DNA damage response protein kinase Rad53 (hChk2) is essential for an mtDNA inheritance checkpoint [82]. In mtDNA-depleted rho° cells, the DNA helicase Pif1 (petite integration frequency 1) undergoes Rad53-dependent phosphorylation. Pif1 is a highly conservative helicase localized to both nucleus and mitochondria in yeast and human cells [83] and promotes DNA replication through interaction with G-quadruplex DNA sequences ([84], reviewed in [85]). Thus, loss of mtDNA activates a nuclear checkpoint kinase that inhibits G1- to S-phase progression [82]. Pif1 is only one example of nuclear DNA helicases to protect mtDNA but, notably [86], it also has an essential role in recombination-dependent replication (as discussed subsequently). Future research may lead to the identification of other factors involved in the crosstalk between nuclear and mitochondrial genome duplication, and even improve our understanding of how the control of mitochondrial replication initiation is related to genome stability, aging, and mitochondrial diseases.

## 2. Origin-Independent Replication

### 2.1. Break-Induced Replication 

**Figure 2:**
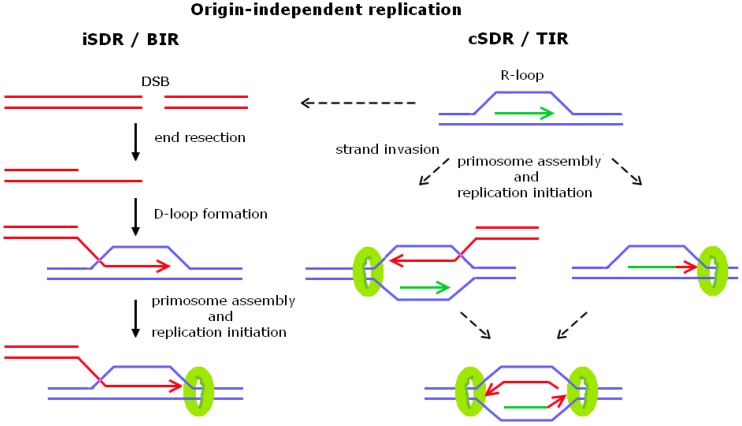
Schematic representation of possible mechanism involved in origin-independent replication initiation by inducible stable DNA replication/break-induced replication (iSDR/BIR) or constitutive stable DNA replication/transcription-initiated replication (cSDR/TIR). Invading and newly synthesized DNA (red), RNA (green), and helicases (green circle) are indicated. Dashed arrows indicate putative scenarios for TIR-dependent replication initiation. Note that none of these scenarios have been experimentally verified. See text for more details. DSB: double-strand break.

A classic example of the initiation of origin-independent DNA replication events is recombination-dependent DNA replication, often called break-induced replication (BIR; see Figure 2 and Table 2, and [87] for a review). Kogoma and colleagues originally designated BIR in bacteria as DNA damage-inducible DNA replication, termed inducible stable DNA replication ((iSDR) [88,89], and reviewed in [90]). Double-strand end repair is initiated by break recognition and loading of the RecBCD helicase/nuclease complex. DNA unwinding by RecBCD leads to subsequent binding of RecA to ssDNA. Then, the strand exchange reaction between two recombining DNA double helices was proposed to as the mechanism by which DNA replication is primed [91,92]. DnaA is essential for helicase loading at *oriC*, whereas PriA, PriB, PriC, and DnaT appear to load DnaB into the forming replisome to promote replication fork assembly at a recombinational D-loop structure ([93], see [94] for a review). Finally, the branch migration and Holliday-junction resolving activities of the RuvABC complex are involved in the resolution of converging replication intermediates generated during iSDR [95].

BIR was later found to occur in yeast upon transformation of yeast with linearized DNA fragments [96,97]. BIR turned out to promote DNA replication restart at broken replication forks and telomeres ([98,99], and reviewed in [87,100,101]) being an error-prone recombination-dependent DNA repair process that occurs in G2/M when only one end of a double-strand break (DSB) is available for recombination [102]. BIR can be Rad51-dependent or independent [102,103]. Rad51 is homologous to the bacterial ssDNA-binding protein RecA, and mainly involved in the search for homology and strand-pairing stages of homologous recombination [104]. Rad51-independent BIR at a one-ended break can occur when long-range strand invasion is not required. It primarily operates during intramolecular recombination; however, intermolecular events mostly rely on Rad51-dependent strand invasion [98,105]. More than 95% of BIR events in *S. cerevisiae* are reported to be Rad51-dependent and do not require either Rad50 or Rad59 [98,106], thus we discuss the Rad51-dependent pathway in more detail. During Rad51-dependent BIR, a DSB end is resected to produce a 3′-ended single-stranded DNA tail, subsequently coated by Rad51 nucleoprotein filaments [102]. This Rad51 filament then invades a homologous sequence and a D-loop is created, followed by an extension of the invading strand by new DNA synthesis using the paired homologous sequence as a template [107]. BIR is known to be a multistep process in which strand invasion occurs rapidly; by contrast, new DNA synthesis does not initiate until 3–4 h after strand invasion [99,102,108]. Once initiated, DNA synthesis may be very processive and continue to the end of the donor chromosome (reviewed in [109]).

Yeast proteins taking part in BIR also play a role in recombination. Recombination proteins Rad51, Rad52, Rad54, Rad55, and Rad57 initiate BIR by promoting strand invasion and D-loop formation [88,98]. BIR requires leading- and lagging-strand DNA synthesis and all essential DNA replication factors, including Pol-α-primase,Cdc7,Cdt1, Mcm10, Ctf4 and CMG helicase complex (except Cdc6 and ORC proteins), specific for pre-RC assembly and specifically needed for origin-dependent DNA replication [99,110]. It still remains to be determined how MCMs are recruited to the D-loop, but it is important to note that BIR occurs at the G2/M phase and normally depends on the Pif1 helicase. BIR may initiate in the absence of Pif1, but Pif1 appears to be required for long-range synthesis during BIR that proceeds by asynchronous synthesis of leading and lagging strands and leads to conservative inheritance of the new genetic material [111,112]. Analysis of BIR-dependent replication intermediates by 2D-agarose gels [113] revealed bubble arc-like migrating structures suggesting the accumulation of ssDNA at unrepaired DNA lesions within the template strand [112,114]. Investigation of BIR in yeast diploid cells led to observation of frequent switches of BIR between two homologous DNA templates, leading to the proposal that BIR is initiated via an unstable replication fork [115]. It was proposed that BIR could occur by several rounds of strand invasion, even at dispersed repeated sequences [115], leading to chromosome rearrangements [116]. However, the specific mechanisms of multiple strand invasions, D-loop displacement, and transition to a stable replication fork remain unknown.

Pol32, a nonessential subunit of Pol-δ, is another key player in BIR [111]. Pol32’s role in BIR is not unequivocally clear, but it has been reported to be essential for Rad51-dependent BIR [99] and required for replication fork processivity [111]. Interestingly, it has been recently shown that theMus81 endonuclease is required to limit BIR-associated template switching during Pol32-dependent DNA synthesis [117]. The involvement of structure-specific nucleases in BIR, such as Mus81-Mms4, Slx1-Slx4, and Yen1, suggests that these nucleases are needed for the processing or resolution of various types of BIR-dependent replication intermediates [118].

The establishment of a replication fork appears to be the slowest step in BIR. In bacteria, the normal initiation role of the DnaA and DnaC proteins in loading DnaB helicase at origins is replaced by the PriA complex (reviewed in [119,120]). PriA is implicated in loading DnaB onto replication fork structures other than replisomes, thus making PriA indispensable for the completion of any replication fork repair [121]. There is no obvious PriA homologue in eukaryotes, but it has been speculated that such a protein must exist. In yeast, the DnaB helicase function is provided by the Mcm2–7complex, which is conserved in all eukaryotes. The Cdc7–Dbf4 protein kinase promotes assembly of a stable Cdc45–MCM complex exclusively on chromatin in S phase [37], and, interestingly, BIR also requires the cell cycle-dependent kinase Cdc7 to initiate BIR [110]. As Rad51-dependent BIR occurs efficiently in G2-arrested yeast cells [102], either a subset of replication-competent MCM helicases remain bound to already replicated DNA, or DNA damage signaling leads to MCM-complex loading and Cdc7-dependent BIR activation in G2 phase. Recent studies show that SUMOylation and polyubiquitylation of MCM proteins have a role in replication initiation and termination, respectively [122,123,124]. It still remains to be determined if these post-translational MCM modifications affect BIR and if other helicases can drive BIR in the absence of MCM proteins. Pif1 may do so, as it already has a known role in BIR [111]. Pif1 is phosphorylated in response to DNA breaks by the Mec1/Rad53 DNA damage pathway in order to block the activity of telomerase at DNA breaks but not at chromosome ends [125], and its phosphorylation is required for BIR-mediated telomere replication in yeast [126]. Although this is pure speculation, it is conceivable that Pif1 might also be prone to Cdc7-dependent phosphorylation in order to fulfill its function in recombination-coupled DNA synthesis.

### 2.2. Transcription-Initiated Replication

R-loops have been shown to have roles in T4 bacteriophage, *E. coli* ColE1 plasmid, and mtDNA replication as well as B-cell immunoglobulin class switch recombination. R-loops are abundant structures, however, unscheduled R-loop formation challenges genome dynamics and function [127,128], and is related to neurological diseases and cancer (reviewed in [129,130,131,132,133]).

The role of R-loops in replication initiation was first demonstrated in *E. coli* ColE1 plasmid [134,135,136] and bacteriophage T4 replication (reviewed in [137]). Another legacy of Tokio Kogoma and colleagues was the discovery of *oriC*-independent DNA replication events ([138,139,140], reviewed in [90]). This type of replication was named constitutive stable DNA replication (cSDR) and, surprisingly, *E. coli* cells can stay alive exclusively on these origin-independent initiation events. One mutation that conferred this phenotype was found to inactivate the *rnhA* gene encoding RNase H1, an RNase specific to RNA in the RNA:DNA hybrid form [141,142]. cSDR was thought to originate from chromosomal sites named *oriK*, and only recently have specific candidate locations for *oriK* been mapped [143]. Moreover, it has been shown that origin-independent DNA synthesis arises in *E. coli* cells lacking the RecG helicase and results in chromosome duplication [144]. In contrast to RNase H1, RecG deals with replication fork fusion intermediates [145,146]; hence, origin-independent synthesis is initiated in different ways, but in both cases a fraction of forks will proceed in an orientation opposite to normal [144]. Drolet et al. [147] provided first evidence that R-loops can accumulate incells lacking *topA*, which encodes a type 1A topoisomerase that relieves negative supercoiling behind the RNAP, by showing that overexpression of *rnhA* partially compensates for the lack of *topA*. Notably, *E. coli* possesses two type 1A enzymes, Top1 (*topA*-encoded) and Top3 (*topB-*encoded), but only cells lacking Top1 are prone to cSDR [148]. Apart from transcription, cSDR requires RecA, and the primosome-complex including PriA, PriB, DnaT, and DNA Pol I [90,149,150]. RecA may also participate in cSDR by binding to ssDNA to stabilize an R-loop, or facilitate an inverse strand exchange reaction performed by RecA ([151,152], see Figure 2). In cSDR, DNA Pol I is thought to extend the RNA of the R-loop and to provide a substrate for PriA binding, as well as DnaB and DNA Pol III loading [90]. Interestingly, cSDR uses the same replicative helicase (DnaB) and replisome components (DNA Pol III) to initiate replication from *oriC*, but uses the PriA-dependent primosome for replicative helicase loading [90], as is the case for replication restart of disassembled replisomes [94]. Improperly regulated DNA replication may lead to various consequences related to genome instability. Interestingly, evidence that R-loop-dependent replication leads to DNA breakage and genome instability in non-growing *E. coli* cells has been presented [153], and mutations reducing replication from R-loops suppress the defects of growth, chromosome segregation, and DNA supercoiling in cells lacking Top1 and RNase H1 activity [154].

Transcription-linked replication initiation in eukaryotic cells was thought to be an exclusive feature of mtDNA replication. Yet, some highly transcribed DNA regions, such as RNAPI-transcribed ribosomal DNA (rDNA) or RNAP III-transcribed genes, were shown to be hot spots for R-loop formation in yeast mutants lacking RNases H [155,156]. In addition, mutants lacking an RNA/DNA helicase Sen1 [157,158] or the yeast Pab1-binding protein Pbp1 (hAtaxin-2) had been found to increase R-loop formation [159]. The absence of RNase H and Top1 activities causes synthetic lethality in yeast, suggesting that persistent R-loop formation could constrain cell viability [160,161]. Accordingly, persistent R-loop formation could be induced by treatment of RNase H mutants with the Top1 inhibitor camptothecin (CPT) leading to the detection of unscheduled transcription-initiated replication (TIR) events in yeast ([161], see Figure 2). TIR initiation intermediates were observed within the rDNA region, but were not linked to a defined replication origin; moreover, they were observed in the late S/G2 phase of the cell cycle, when replication termination and completion was expected to take place [161]. TIR was RNAPI transcription-dependent and led to replication fork pausing sites at sites of protein–DNA interaction. Taken together, these results suggest that R-loops could mediate origin-independent replication initiation events that constitute a non-canonical replisome, lacking the factors required to bypass replication constrains.

The factors and mechanisms participating in transcription-initiated replication events still remain to be elucidated. Various nonexclusive mechanisms could cooperate to trigger TIR events (summarized in Figure 2). These include strand invasion-dependent replication events that might be stimulated by the presence of single-stranded DNA within R-loops. In the absence of RNase H and Top1 activities, the rDNA locus turns into a hotspot for DSBs [161], thus it is conceivable that these DSBs drive recombination-dependent replication such as BIR. Other possibilities include that R-loops cause replication fork collapse and TIR is the result of replication restart of a replisome–RNAP complex [162,163]. An interesting possibility would be de novo replisome assembly at an R-loop. The RNA present within the R-loop could prime leading-strand synthesis and provoke assembly of replication-competent replicases at S/G2 phase [164]. Apparently, ssDNA opposite an RNA:DNA hybrid could activate Mec1-mediated checkpoint activation and binding of the replication protein A (RPA) complex, which has been shown to be involved in replication initiation as well as DNA repair by interacting with both the DNA Pol-α-primase complex and with DNA Pol-δ [164,165]. An R-loop may promote DNA replication restart by Pol-α-driven DNA synthesis, since the essential DNA Pol-α-primase subunit Pol12 remains active and phosphorylated in S/G2 and is inactivated while cells exit mitosis [44,161,166]. Moreover, a recent work by Symington and coworkers suggests that BIR occurs by a conservative mode of DNA synthesis [107]. Thus, it will be interesting to determine whether the same is true for TIR, or if TIR pursues a semiconservative replication mode. It is striking that in *E. coli*, many factors involved in iSDR are also needed for cSDR. These findings suggest that in yeast, many factors involved in BIR might be required for TIR. These factors include proteins involved in homologous recombination, DNA end-processing, helicases, primases, DNA polymerases, and, finally, structure-specific endonucleases (as listed in Table 2). Nevertheless, genetic interactions in yeast cells between RNase H deficiencies and proteins involved in BIR still remain to be determined.

Yet-to-be determined questions include whether TIR is limited to rDNA, and whether TIR can be observed in other RNA/DNA helicases mutants, including Sen1 [156,157,158] or the yeast ataxin-2 protein Pbp1 [159]. Recently, it has been shown that replication initiates, albeit very infrequently, within the telomeric repeats [167]. A long noncoding telomeric repeat-containing RNA (TERRA) has been implicated in telomere maintenance during replicative senescence and cancer [168,169]. TERRA accumulates specifically at short telomeres and may promote replication-fork restarting by recruiting homology-directed repair (HDR) mediators or even by directly priming replication in an origin-independent manner [167], similar to what was reported by Stuckey et al. [161]. This proposal might be supported by the fact that the cell cycle regulation of TERRA becomes perturbed at telomeres that are maintained by HDR, and that TERRA remains telomere-associated at G2/M in cells that use the alternative lengthening of telomeres (ALT) mechanism [170]. Interestingly, loss of ATP-dependent helicase ATRX that is frequently mutated in ALT-positive cancers, leads to persistent association of RPA with telomeres after DNA replication [170]. ATRX is involved in establishing transcriptionally silenced heterochromatin, and one hypothesis is that ATRX helicase and ATPase activity resolves G4 DNA secondary structures formed opposite of a TERRA-containing R-loop ([169,171], reviewed in [167]).

## 3. Conclusions

Since the detection of recombination-dependent replication of the *E. coli* chromosome by Lark and Kogoma about 50 years ago [172], we have learned a lot about mechanisms that can lead to non-canonical replication initiation in prokaryotic and eukaryotic cells. It is generally accepted that recombination serves to rescue broken chromosomes and stalled replication forks, however, we are far away from the complete picture on how cells manage to bypass the need for origin-dependent replication initiation. The mechanistic models and enzymatic steps leading to iSDR and cSDR in *E. coli* can be considered as a blueprint for BIR and TIR events in eukaryotic systems. Interestingly, all known features of BIR and TIR can participate in mtDNA replication events. Nevertheless, an important difference is noted by the fact that nuclear BIR and TIR events happen in a chromatin context with eukaryotic replication, starting with nucleosome packaging.

Many aspects of non-canonical DNA replication in eukaryotes still remain unknown and deserve to be addressed in the future; in particular, the factors driving replication fork progression and the mode of TIR-dependent DNA synthesis need to be characterized. Special attention should be given to the identification of key replication factors involved in TIR, such as DNA polymerases and helicases, but also to otherwise auxiliary replication proteins such as Pol32. R-loops are essential for the onset of TIR, and this might not be the only difference between TIR and BIR events. As outlined in Figure 2, the question remains if TIR is driven by strand invasion of the R-loop. TIR has been characterized only in repetitive ribosomal DNA sequences, raising the question of whether it is sister-chromatid-dependent, or if it uses non-sister chromatids as a template for DNA synthesis. In either case, strand invasion could be Rad51-dependent or independent. However, the role of Rad51 in TIR still needs to be determined. Genetic screens might help to shed light on factors required for TIR initiation and provide more insight to the differences between TIR and BIR.

The other model proposed in Figure 2 includes de novo assembly of a replication fork at an R-loop. In this case, which replication factors would be assembled at an R-loop, and would this kind of non-canonical replication restart be S phase-dependent? Would conservative or semiconservative replication account for the newly synthesized DNA? Could an R-loop even contribute to the activation of less defined replication origins in higher eukaryotes? Unrevealed functions of R-loops in higher eukaryotes may include a role the epigenetic regulation of origin-dependent replication initiation [173,174]. Interestingly, a nuclease-resistant G-quadruplex hybrid structure involving both RNA and DNA is present at the mtDNA replication initiation site [65]. G-rich RNA mediates Epstein-Barr virus nuclear antigen 1 EBNA1 and ORC interaction [175], thus it is conceivable that that transcription-related RNA structures might replace the need for specific origin-recognition sequences. By using a high-resolution PCR strategy to localize replication origins directly on total unfractionated human DNA, over-replicated regions were found to overlap with transcription initiation sites of CpG island promoters [176] and, recently, active transcription was proposed to be a driving force for the human parasite *Leishmania major* spatial and the temporal program of DNA replication [177]. Last but not least, TIR could be considered as an ancient mechanism to promote gene amplification events linked to nuclear differentiation and evolution. In order to resolve these questions, future studies should include higher eukaryotic model systems to see if TIR has a role in genome stability connected to various human diseases, including cancer.

## Figures and Tables

**Table 1:** Factors required for origin-dependent DNA replication initiation in *Escherichia coli* and *Saccharomyces cerevisiae.*

Origin-Dependent Replication	*E. coli*	*S. cerevisiae*	
	Chromosomal DNA Replication	Chromosomal DNA Replication	Mitochondrial DNA Replication
Origin	*OriC*	ARS	*OriH*, *OriL*
DNA unwinding	DnaA, DnaB, DnaC, SSB	Cdc45, GINS, Mcm2–7, Mcm10, RPA	Rpo41, Irc3, Rim1
Replication priming/elongation	DnaG, DNA Pol III	DNA Pol-α-primase, DNA Pol-ε and Pol-δ	Rpo41, DNA Pol-γ

SSB: single-stranded DNA-binding protein; DNA Pol: DNA polymerase; RPA: replication protein A; ARS: autonomously replicating sequence.

**Table 2:** Factors required for origin-independent DNA replication by iSDR/BIR or cSDR/TIR.

***E. coli***	**Function**	**iSDR**	**cSDR**
	End processing	RecBCD	RecBCD
	Strand invasion	RecA	RecA
	DNA unwinding	DnaBC, PriAB	DnaBC, PriAB
		RecG	?
		DnaT	?
	Replication priming/elongation	DnaG, DNA Pol III	DnaG, DNA Pol I/Pol III
	Resolution	RuvABC	?
***S. cerevisiae***	**Function**	**BIR**	**TIR**
	End processing	MRX (Mre11-Rad50-Xrs2)	?
	Strand invasion	Rad51*, Rad52, Rad54, Rad55, Rad57	?
	DNA unwinding	Cdc45-MCM-GINS, DDK, Mcm10, Ctf4, RPA, Pif1	RNA:DNA hybrid
	Replication priming/elongation	Pol-α-primase, Pol-δ, Pol32*	?
	Resolution	Mus81-MMS4, Slx1–Slx4, Yen1	?

Note that BIR can be Rad51 and/or Pol32 independent (*). MCM: minichromosome maintenance complex; DDK: Dbf4-dependent kinase; Pif1: petite integration frequency 1.
